# Supplementary material for: Drought resistance of ten ground cover seedling species during roof greening
Source: PLoS One. 2020 Jun 24;15(6):e0220598. doi: 10.1371/journal.pone.0220598 (PMC7313738; doi:10.1371/journal.pone.0220598)
Supplement: Attachment — Submitted filename: 4.Response to Reviewers.docx [file pone.0220598.s002.docx]

Dear,

Dr. Jamie Males.

I appreciate your attention to the manuscript.

We have edited the manuscript very carefully in this version:

Firstly, we revised the grammar and spelling of the manuscript completely that included tense, voice, syntactical structure, and some spelling mistakes.

Secondly, we deleted some words that had nothing to do with the manuscript itself.

In addition, we removed funding information from the manuscript “this work was supported by the Young Core Plan of the Beijing Academy of Science and Technology (BJAST) (No. 201528), the Beijing Natural Science Foundation (No. 8142017) and the National Natural Science Foundation of China (No. 41475133)” and add them in cover letter.

Kind regards,

Dr. Yanju-Liu
