# Supplementary material for: Drought resistance of ten ground cover seedling species during roof greening
Source: PLoS One. 2020 Jun 24;15(6):e0220598. doi: 10.1371/journal.pone.0220598 (PMC7313738; doi:10.1371/journal.pone.0220598)
Supplement: Attachment — Submitted filename: Reviewers Comments.docx [file pone.0220598.s003.docx]

Title: Drought Resistance of 10 Ground Cover Seedling Species during Roof Greening from Zhang et al. which was submitted to PloS One has meaningful results and well conducted experiment. The use of different plant species i.e. Paeonia lactiflora, Hemerocallis dumortieri, Physostegia virginiana, Iris lacteal, Hylotelephium erythrostictum, Sedum lineare, Iris germanica, Cosmos bipinnata, Hosta plantaginea and Dianthus barbatus for determining the drought tolerant and drought sensitive species is a novel research. I recommend this article should be publish in PloS One before changing some minor mistakes which are as follow:

On the abstract: You have mentioned Cosmos bipinnata and Physostegia virginiana died while other plant species survived. I recommend you please change the terminology like died is not suitable for the plant you can write in a better way.

On the introduction: The information regarding ROS or oxidative stress is too limited. You should add at least 2-3 sentences about their mechanism, effect and actions of antioxidants in response to oxidative stress. <https://doi.org/10.1016/j.ecoenv.2019.109915>. doi:10.3390/plants8120545. <https://doi.org/10.1007/s11356-019-07264-7>. Please see these latest articles which just published recently and find some relevant information about your topic and cites these in introduction.

On Material and Method: You have mentioned “The seedlings were provided by the Yu-quanying flower market, a large and popular market in Beijing” can you provide more details about it (if possible).

You have mentioned “mg·kg-1” Please see above mention articles and see how we can correctly write this unit and write correctly throughout your manuscript.

You have mentioned “Leaves were placed into sealed plastic bags and kept in a portable ice box at 0 ~ 4℃” Scientifically when you are collecting your samples please took in liquid nitrogen. You can taken in the ice box. It’s ok but more convenient to take in liquid nitrogen.

On Results and discussions: Better to write chl a like this: use first time full abbreviation.

“Drought stress causes changes in chlorophyll content in plants” Please modify this sentence.

You have find that “The chlorophyll content of Physostegia virginiana was the highest, at 49.07 mg/g·FW” This is the maximum chlorophyll contents and determined by Arnon’s (1949) method. Are you sure that you determined too much chlorophyll in this plant species. For the best of our knowledge we did not saw too much high chlorophyll contents. Please verify your formula or your method and make sure that chlorophyll is very high? Please see it carefully.

“The chlorophyll content of Hosta plantaginea was the highest, at 59.11 mg/g·FW” Is it?

Please verifily the units of antioxidants you have determined “U·min-1 ·g -1 FW” or “U·mg-1” Please make sure that you are going through with right units.
