# Supplementary material for: Drought resistance of ten ground cover seedling species during roof greening
Source: PLoS One. 2020 Jun 24;15(6):e0220598. doi: 10.1371/journal.pone.0220598 (PMC7313738; doi:10.1371/journal.pone.0220598)
Supplement: Attachment — Submitted filename: Response to Reviewers.doc [file pone.0220598.s004.doc]

**Response to Reviewers**

First, we are very grateful to the reviewers for their careful work and useful advice, which are truly beneficial for improving the quality of the article. We have accepted the comments of the reviewers and editor and have tried our best to revise the article as needed.

- **Response to Reviewer #1’s main comments:**

**Summary comment:** The section of results and discussion required a “Major Revision”.

**Summary response:** Yes. Thanks for review’s nice suggestions. We have revised the manuscript according to each of the comments as follows.

**Comments 1:** The explain of MP, Chl, SOD, POD, and AsAPOD(APX) means required greater depth.

**Response 1:** We looked up a large number of papers and cited 31 of them to elaborate further on the importance of the MP, Chl, SOD activity, POD activity, and APX activity indicators. Not only was the first paragraph of each section (section 4.2 to 4.6) supplemented with explanations of the indicators but also some scientific discussions were interspersed throughout other paragraphs.

**Comments 2:** The describes of statistical differences among species is not enough, it needs further explain why species have such change under the watering condition.

**Response 2:** We have strengthened the discussion of each indicator. On the basis of the objective elaboration of the statistical data, an interpretation of the changes in the data has been added.

For instance, in section 4.2, we further explained the cause of death of *Cosmos bipinnata* and *M. urticifolia* under drought stress, explained the reason why *C. bipinnata* presented increased MP values and further explained the why the stability of the various membrane systems of the cells means strong resistance to drought stress.

In section 4.3, we first summarized the general trends of plant Chl before the detailed discussion. We added a paragraph to discuss the changes in different species from the family perspective, which made the discussion are more reasonable.

Similarly, we have made in-depth revisions to section 4.4. We summarized three suggestions: (1) drought stress induced an increase in SOD activity, and by increasing their SOD activity, the abovementioned plant species eliminated ROS; (2) the plant species presenting increased SOD activity showed advantages in terms of their drought stress response; and (3) proper drought exposure could significantly improve plant resistance to sustained drought stress.

We also strengthened the link among the POD, APX, and SOD protective enzymes in the following two sections. We found that there may be a complementary relationship among POD, APX, SOD. On the basis of the change trends of the activity of these three protective enzymes, SOD may play a dominant role in scavenging ROS during severe drought stress (or during the late period of drought stress), and APX and POD may play a dominant role in scavenging ROS during moderate drought stress (or during the early period of drought stress).

In accordance with the reviewer's suggestion, we added details to Table 3 (the moisture content of the soil and the increases in soil RWC on the sampling day).

**Comments 3:** Figures 4 and 5 may improve their readability, rather than using broken y-axes.

**Response 3:** We improved the figures in the paper. At first, “Cg”, “T1” and “T2” were used instead of “well-watered”, “moderate” and “severe”, respectively. Secondly, Figures 4 and 5 have been changed into log-scale figures.

- **Response to Reviewer #2’s comments:**

**Comments 1:** On the abstract: You have mentioned *Cosmos bipinnata* and *Physostegia virginiana* died while other plant species survived. I recommend you please change the terminology like died is not suitable for the plant you can write in a better way.

**Response 1:** We used the word “is not suitable for” instead of “died”.

**Comments 2:** On the introduction: The information regarding ROS or oxidative stress is too limited. You should add at least 2-3 sentences about their mechanism, effect and actions of antioxidants in response to oxidative stress.

**Response 2:** Following reviewer’s suggestion, we not only supplemented some descriptions of ROS but also added many descriptions to other parts of the discussion.

**Comments 3:** On Material and Method: You have mentioned “The seedlings were provided by the Yu-quanying flower market, a large and popular market in Beijing” can you provide more details about it (if possible).

**Response 3:** We have added some a specific description of the market.

**Comments 4:** You have mentioned “mg·kg^-1^” Please see above mention articles and see how we can correctly write this unit and write correctly throughout your manuscript.

**Response 4:** We checked the reference of “ZHU Minghao, et al. The impact of *Elaphurus davidianus* in different habitats on soil physical and chemical properties [J]. Environmental Chemistry, 2016, 35(1): 208-217. DOI:10.7524/j.issn.0254-6108.2016.01.2015070803”. Again, units of “mg·kg^-1^” were correct.

**Comments 5:** Better to write chl a like this: use first time full abbreviation.

**Response 5:** We have replaced “Chl a” by “Chlorophyll a” when it appeared first time.
