# Supplementary material for: Drought resistance of ten ground cover seedling species during roof greening
Source: PLoS One. 2020 Jun 24;15(6):e0220598. doi: 10.1371/journal.pone.0220598 (PMC7313738; doi:10.1371/journal.pone.0220598)
Supplement: Attachment — Submitted filename: Response to reviewers.docx [file pone.0220598.s005.docx]

**Response to Reviewers**

We authors appreciate Reviewer #1 and editor for your detailed guide to further revise the manuscript. We have renewed the content following your suggestions.

**Summary of Reviewer #1’s viewpoints:**

Reviewer #1 took the section 4.2 as an example and showed us how to organize the article structure.

According to the suggestions, we made the following major changes in Results and Discussion:

1. Move the first paragraphs in "Result and Discussion" to the “Introduction" section.

2. For each parameter, statistically significant changes were described instead of detailed species data lists.

3. We focued on the important phenomenon and their scientific explanation.

4. We summarised the major finding points in the last paragraph for MP, SOD and POD.

In addition, we corrected the spelling mistakes , grammatical and other errors.
